# What Makes a Quality Health App—Developing a Global Research-Based Health App Quality Assessment Framework for CEN-ISO/TS 82304-2: Delphi Study

**DOI:** 10.2196/43905

**Published:** 2023-01-23

**Authors:** Petra Hoogendoorn, Anke Versluis, Sanne van Kampen, Charles McCay, Matt Leahy, Marlou Bijlsma, Stefano Bonacina, Tobias Bonten, Marie-José Bonthuis, Anouk Butterlin, Koen Cobbaert, Thea Duijnhoven, Cynthia Hallensleben, Stuart Harrison, Mark Hastenteufel, Terhi Holappa, Ben Kokx, Birgit Morlion, Norbert Pauli, Frank Ploeg, Mark Salmon, Kyma Schnoor, Mary Sharp, Pier Angelo Sottile, Alpo Värri, Patricia Williams, Georg Heidenreich, Nicholas Oughtibridge, Robert Stegwee, Niels H Chavannes

**Affiliations:** 1 National eHealth Living Lab Department of Public Health and Primary Care Leiden University Medical Center Leiden Netherlands; 2 Ramsey Systems Ltd Shrewsbury United Kingdom; 3 Assessment Team ORCHA Health Ltd Liverpool United Kingdom; 4 Healthcare Royal Netherlands Standardization Institute Delft Netherlands; 5 Health Informatics Centre Department of Learning, Informatics, Management and Ethics Karolinska Institutet Stockholm Sweden; 6 Privacy1 B.V. Groningen Netherlands; 7 Projects Accessibility Foundation Utrecht Netherlands; 8 European Coordination Committee of the Radiological, Electromedical and Healthcare IT Industry Brussels Belgium; 9 Quality, Standards and Regulations Innovation and Strategy Philips Belgium Commercial N.V. Brussels Belgium; 10 Understandable Information and Digital Healthcare Pharos - Dutch Center of Expertise on Health Disparities Utrecht Netherlands; 11 ETHOS Ltd Exeter United Kingdom; 12 Institute for Software Technology and Data Communication Hochschule Mannheim Mannheim Germany; 13 Technical Committee 251 Health informatics European Committee for Standardization Brussels Belgium; 14 USBIMED Oulu Finland; 15 Product Security Group Security Philips Electronics Nederland B.V. Best Netherlands; 16 Unit eHealth, Well-Being and Ageing Directorate‑General for Communications Networks, Content and Technology European Commission Luxembourg Luxembourg; 17 Draeger Integrated System Management Drägerwerk AG & Co. KGaA Lübeck Germany; 18 Mobile Health Work Group Health Level Seven Brussels Belgium; 19 Enterprise Architecture University Medical Center Groningen Groningen Netherlands; 20 Science, Evidence and Analytics Directorate National Institute for Health and Care Excellence Manchester United Kingdom; 21 School of Computer Science and Statistics Trinity College Dublin Ireland; 22 Technical Committee 527 Health informatics Ente Italiano di Normazione Milan Italy; 23 Mexedia S.p.A. SB Rome Italy; 24 Faculty of Medicine and Health Technology Tampere University Tampere Finland; 25 Flinders Digital Health Research Center College of Science and Engineering Flinders University Adelaide Australia; 26 Technical Committee 215 Health informatics International Organization for Standardization Geneva Switzerland; 27 Subcommittee 62A Common aspects of medical equipment, software, and systems International Electronical Commission Geneva Switzerland; 28 Healthcare IT Standards Siemens Healthcare GmbH Erlangen Germany; 29 NHS Transformation NHS England Leeds United Kingdom

**Keywords:** health app, wellness app, mobile health, mHealth, Delphi technique, quality assessment, assessment framework, standard, standardization, COVID-19

## Abstract

**Background:**

The lack of an international standard for assessing and communicating health app quality and the lack of consensus about what makes a high-quality health app negatively affect the uptake of such apps. At the request of the European Commission, the international Standard Development Organizations (SDOs), European Committee for Standardization, International Organization for Standardization, and International Electrotechnical Commission have joined forces to develop a technical specification (TS) for assessing the quality and reliability of health and wellness apps.

**Objective:**

This study aimed to create a useful, globally applicable, trustworthy, and usable framework to assess health app quality.

**Methods:**

A 2-round Delphi technique with 83 experts from 6 continents (predominantly Europe) participating in one (n=42, 51%) or both (n=41, 49%) rounds was used to achieve consensus on a framework for assessing health app quality. Aims included identifying the maximum 100 requirement questions for the uptake of apps that do or do not qualify as medical devices. The draft assessment framework was built on 26 existing frameworks, the principles of stringent legislation, and input from 20 core experts. A follow-up survey with 28 respondents informed a scoring mechanism for the questions. After subsequent alignment with related standards, the quality assessment framework was tested and fine-tuned with manufacturers of 11 COVID-19 symptom apps. National mirror committees from the 52 countries that participated in the SDO technical committees were invited to comment on 4 working drafts and subsequently vote on the TS.

**Results:**

The final quality assessment framework includes 81 questions, 67 (83%) of which impact the scores of 4 overarching quality aspects. After testing with people with low health literacy, these aspects were phrased as “Healthy and safe,” “Easy to use,” “Secure data,” and “Robust build.” The scoring mechanism enables communication of the quality assessment results in a health app quality score and label, alongside a detailed report. Unstructured interviews with stakeholders revealed that evidence and third-party assessment are needed for health app uptake. The manufacturers considered the time needed to complete the assessment and gather evidence (2-4 days) acceptable. Publication of CEN-ISO/TS 82304-2:2021 *Health software* – *Part 2: Health and wellness apps* – *Quality and reliability* was approved in May 2021 in a nearly unanimous vote by 34 national SDOs, including 6 of the 10 most populous countries worldwide.

**Conclusions:**

A useful and usable international standard for health app quality assessment was developed. Its quality, approval rate, and early use provide proof of its potential to become the trusted, commonly used global framework. The framework will help manufacturers enhance and efficiently demonstrate the quality of health apps, consumers, and health care professionals to make informed decisions on health apps. It will also help insurers to make reimbursement decisions on health apps.

## Introduction

### Background

Health apps include “wellness apps” (eg, targeting physical activity and diet) and “medical apps” (eg, diagnosing and monitoring conditions) [1]. Given their role in enhancing individual health, increasing work productivity, and reducing work absence, the potential of these apps has been estimated at 99 billion euros in health care cost savings for European health care systems and citizens in 2017 alone, and another 93 billion euros contribution to the gross domestic product and income taxes [2]. In addition to this financial impact, the European Commission’s Digital Single Market strategy also highlights the potential benefits of digital health in addressing unequal quality of and access to health care services, as well as the shortage of health professionals [3]. Most recently, the COVID-19 pandemic highlighted the rapid and sustained response potential of health apps. Health apps were deployed to offer trustworthy information, discover predictive symptoms, trace contacts, provide proof of vaccination or testing, address physical and mental health, and maintain and relieve regular patient care via digital consultations and remote monitoring [4-6].

Despite this great potential, uptake of health apps has been slow [7,8]. Adoption barriers include a lack of clarity about certification, a lack of benefit awareness, and a lack of reimbursement mechanisms [2]. Moreover, there is no consensus on what makes a high-quality health app [9]. Similarly, there is no efficient, transparent, and widely adopted assessment process or accessible expression of assessment results, for instance in the form of an internationally recognized label [10-12]. Standardization involving all stakeholders has been advocated [13-15] in order to guarantee app quality, mitigate risks, assist app development, enable informed decisions, and promote uptake in care pathways, pandemic response plans, and reimbursement [16-18].

To address the adoption barriers, the European Commission commissioned the European Committee for Standardization (CEN-CENELEC) to develop common principles for health apps. In line with the CEN/TC 251 business plan, collaboration was immediately sought with the International Organization for Standardization (ISO) and the International Electrotechnical Commission (IEC), making the initiative a global activity. The initiative is at the heart of the European Commission’s Digital Single Market strategy, which aims for person-centered health care and citizen empowerment using digital tools and data. Common European principles and certification are expected to increase the uptake of digital tools by providers and authorities and enable more efficient public funding of these tools.

### Objective

This study aimed to develop, with relevant stakeholders, a useful, trustworthy, and usable health app quality assessment framework with the potential to become the preferred European and global framework.

## Methods

### Study Design

**Figure 1:**
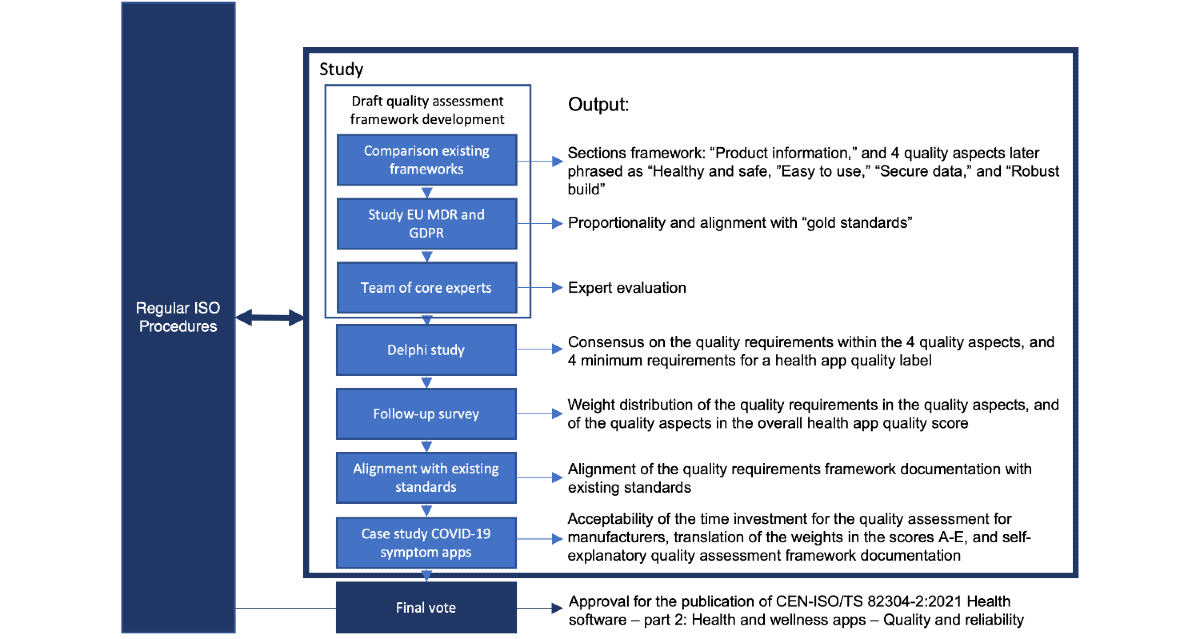
Study phases. EU: European Union; GDPR: General Data Protection Regulation; ISO: International Organization for Standardization; MDR: Medical Device Regulation; TS: technical specification.

The study was undertaken in 5 phases (Figure 1). Firstly, a draft quality assessment framework was developed. This was followed by a 2-round Delphi process with web-based surveys to find consensus on the draft global health app quality assessment framework. This framework includes a set of questions and related evidence that make the quality of a health app transparent. The Delphi technique is commonly used in standardization efforts in health care and is particularly suited to consult at scale geographically and professionally diverse individual expert opinions. A deliberately selected panel of anonymous experts typically needs 2 to 3 rounds of structured surveys including feedback on the results to achieve consensus [19-21]. Thirdly, a follow-up survey was used to build the mechanism of scoring the questions. All digital surveys employed in this iterative process were completed in a data management platform (Castor Electronic Data Capture). After alignment with existing standards, the resulting assessment framework was tested against existing COVID-19 symptom apps. Finally, the international standards community commented and voted on the framework.

### Draft Quality Assessment Framework Development

First, in May and June 2019, a comparison of the quality criteria of 13 existing health app assessment frameworks was made to assess the overarching common health app quality criteria (Multimedia Appendix 1). This resulted in 5 sections for the draft quality assessment—“Product information” and 4 quality aspects. After testing with people with low health literacy, these quality aspects were indicated as “Healthy and safe,” “Easy to use,” “Secure data,” and “Robust build.”

Second, the fundamentals of the European Union (EU) Medical Device Regulation and General Data Protection Regulation were studied. We aimed to ensure proportionality and alignment of the quality assessment framework with the principles of possibly the most stringent legislation globally with regard to medical applications and data. The envisioned technical specification (TS) spans both health apps that qualify as medical devices and apps that do not. Although the TS does not attempt to determine whether a health app should be regulated as in the case of medical devices, the rationale behind our study of the EU Medical Device Regulation was to learn about its risk-based approach and how to extend it proportionally to apps that are not medical devices. Moreover, apps tend to evolve over time, potentially into a medical device. Some quality requirements, regarding both the product and its development process, are best to be considered from the very beginning of the development process.

Third, 13 additional frameworks (Multimedia Appendix 1) and a team of 20 core experts (Multimedia Appendix 2) were consulted in drafting an initial version of the quality assessment framework. This initial version consisted of 116 questions and 22 requests for evidence to enable third-party quality assessment. Unstructured interviews with stakeholders, including health care professional organizations and insurers, revealed that evidence requests and third-party assessments are important preconditions for the uptake of health apps.

### Delphi Study Participant Recruitment

The 2-round Delphi technique was used to ensure that the quality assessment framework contained no less and no more than the required questions for the uptake of health apps. We aimed to involve at least 40 gender-diverse participants from all 6 main continents, representing both the key stakeholders and experts in health app quality assessment. Participants were suggested by international ISO experts involved in developing the TS and also found by searching the internet and literature. Potential participants were invited to participate via email or LinkedIn.

### Delphi Round 1

In round 1 (February 2020), participants were asked to rate the importance of the draft questions and evidence requests that matched their expertise. The response options for all questions and evidence requests ranged from useless (1) to crucial (7) on a 7-point Likert scale. A median of 6 or higher was considered consensus and reason to retain the question or evidence request in the quality assessment framework.

Participants who rated the importance of a question or evidence request at a 1 or 2 were asked to describe their perspective and what they would need to go along with the decision, should the median for that draft question or evidence request be 6 or more. This phrasing was adopted from the Lewis method of deep democracy, developed for consensus building in post-Apartheid South Africa [22]. Participants were able to make editorial suggestions and propose new questions or evidence requests. The opposing perspectives and editorial suggestions were addressed with the help of the core experts. A maximum of 100 questions, preferably less, were suggested to positively affect usability, buy-in, and focus of app manufacturers and the efficiency of the health app quality assessment framework.

### Delphi Round 2

In round 2 (April 2020), participants rated and commented on the 24 new questions and 3 new evidence requests that emerged from round 1 using a similar methodology as in round 1. Participants were again asked to comment on wording, notes, and response options. Suggested changes were discussed with the related core experts.

Inspired by the EU Energy label’s scoring mechanism and minimum requirements, participants were also asked what they would consider an adequate score if the quality requirement was not met. Could the maximum score within the related quality aspect still be a “green A” representing the best score, or should it be a “light green B,” “yellow C,” “orange D,” the worst score, “red E,” or not acceptable (“black F”)? If the median was a “black F” the question was considered a minimum requirement to qualify for the simultaneously developed health app quality label (Multimedia Appendix 3).

### Follow-up Survey

A follow-up survey (June 2020) was used to build the scoring mechanism for communicating the quality assessment results in the health app quality label. The main question asked for “Healthy and safe” and “Secure data” was, “Which approximately 5 requirements should be most significant?” For “Easy to use” and “Robust build,” the question was, “Which approximately 3 requirements should be most significant?” as these quality aspects involve a smaller number of quality requirements. We aimed to reach 3 to 6 organizations from 6 different stakeholder groups, comprising app assessors (including health technology assessment bodies and app stores), app manufacturers, health care authorities, medical or health professional organizations, patient and consumer organizations, and insurers.

### Alignment With Existing Standards

As a next step, the resulting quality assessment framework was aligned with existing standards, for instance, by adding excerpts of these standards in the notes to explain the terminology used and to provide further guidance. The 28 standards referred to in the quality assessment section of the TS are included in Multimedia Appendix 4.

### Case Study COVID-19 Symptom Apps

After aligning with existing standards, the quality assessment framework was tested and fine-tuned by evaluating COVID-19 symptom apps for the Dutch Ministry of Health. Fifteen app manufacturers, as identified by the Dutch Ministry of Health, were invited to participate in a third-party assessment of their COVID-19 app using the draft framework. For inclusion criteria, see Multimedia Appendix 5. All manufacturers were offered an individual 30- to 60-minute telephone call with the principal investigator to resolve any lack of clarity encountered in working with the draft quality assessment framework.

### ISO Procedure

Parallel to the above methods, 61 ISO experts from 14 countries spanning 4 continents produced 4 working drafts of the TS, for which the quality assessment framework became the core content. Experts from 52 countries spanning 6 continents, participating in ISO technical committee (TC) 215, CEN/TC 251, and IEC subcommittee 62A, were invited to submit comments to each of the working drafts [23]. Between March and May 2021, these countries were asked to cast their vote for the final draft of the TS. A simple majority (>55% or 66.7%) vote from the ISO, IEC, and CEN-CENELEC participating members sufficed for publication [24,25].

### Ethics

Ethical approval by the Medical Ethics Committee was not required. All participants were asked for consent at the beginning of the Delphi surveys. The follow-up survey specified the intended use of the responses and that participation implied consent. Standardization experts participated in the ballot and review process of the standard development as part of their membership of the national Standard Development Organization mirror committees. All data were handled following European data protection regulations.

## Results

### Participant Characteristics

The first Delphi survey was sent to 197 stakeholders and experts, the second to an additional 14, adding up to a total of 211. Response was defined as rating the importance of at least one framework question. The response rates per round were 33.5% (66/197) and 27.5% (58/211), respectively. A total of 41 respondents participated in both rounds, and 42 in only one round. The follow-up survey had a response rate of 36.8% (28/76). In both Delphi rounds, the most common background of participants was Small and Medium-sized Enterprise or industry representative (9/65 and 8/56) and medical professional or medical organization (9/65, 14% and 7/56, 13%). In the follow-up survey, health care authority (7/28, 25%) and medical or health professional organization (5/28, 18%) were most common. Five continents were represented in the first Delphi round, and 6 in the second round. Most participants resided in Europe (53/65, 82%, in round 1 and 48/56, 86% in round 2). For more details, see Multimedia Appendix 6.

### Delphi Study

The number of respondents who rated the importance of the proposed quality assessment elements is detailed in Table 1.

Multimedia Appendix 7 lists the questions and evidence requests whose importance was rated at a median of less than 6. They were removed from the quality assessment framework unless the suggestions to rephrase provided a rationale to pose the question differently or a related evidence request had a median of 6 or higher. The draft quality assessment framework that resulted from round 1 consisted of 110 questions and 19 requests for evidence. The 4 questions that had a black F (“not acceptable”) as a median score in round 2 and were thus identified as minimum requirements for the health app quality label are marked with an “R” (for “Required”) in Multimedia Appendix 8.

**Table 1:** Respondents rating the importance of the proposed quality assessment elements.

Number of respondents per newly proposed quality assessment element	Round 1, n (mean, SD)	Round 2, n (mean, SD)
Product information	46 to 62 (53.69, 4.01)	48 to 51 (49.5, 2.12)
Healthy and safe	29 to 51 (40.31, 5.79)	20 to 41 (26.25, 5.65)
Easy to use	22 to 44 (33.37, 8.96)	13 to 28 (23.25, 6.94)
Secure data	21 to 40 (29.51, 6.31)	14 to 26 (19.75, 4.92)
Robust build	25 to 33 (28.87, 2.20)	26

### Follow-up Survey

The quality requirements that were rated most important in the follow-up survey (top-3 “Easy to use” and “Robust build,” and top-5 “Healthy and safe” and “Secure data”) received a weight of 3 in the scoring of these quality aspects. The top-10 minimum requirements that resulted from the survey (Multimedia Appendix 9) were also given a weight of 3. The 4 minimum requirements that emerged from the Delphi study do not impact the score; they only affect the qualification for a label. All quality requirement questions without a weight of 3 but with a 50% or more consensus vote of one or more individual stakeholder groups received a weight of 2. All other questions received a weight of 1.

The top-10 minimum requirements consisted of 6 “Healthy and safe,” 1 “Easy to use,” and 3 “Secure data” questions. Slight adjustments to this distribution informed the “Overall health app quality score,” the product of the scores of the 4 quality aspects. “Robust build” was given a weight of 1 in the overall score at the expense of “Healthy and safe” (weight of 5), as ignoring robust build can affect the health app safety. “Easy to use” (weight 1.5) was given an additional weight of 0.5 at the expense of “Secure data” (weight 2.5), considering the importance end users attribute to ease of use [26]. Multimedia Appendix 8 summarizes the weights of the individual quality requirements in the 4 quality aspects and the weights of these quality aspects in determining the “Overall health app quality score.”

### Case Study COVID-19 Symptom Apps

Of the 15 invited primarily Dutch COVID-19 symptom app manufacturers, 11 (73%) participated and provided the evidence that enabled assessment of their app. Reasons for not participating included lack of time (n=2, 13%), absence of the initiator (n=1, 7%), and the COVID-19 app being only a temporary initiative (n=1, 7%). App manufacturers who participated in the case study reported they had spent half to a full day per quality aspect to fill out the draft version of the quality assessment framework, answer further context-specific questions, and provide the evidence requested, which they considered acceptable. This time investment was also found acceptable by 7 further small- and medium-sized app manufacturers in Europe (through semistructured interviews).

Based on the experiences in the case study and in order to instill trust in the quality assessment as a driver for the uptake and funding of apps, the project group decided going forward to request evidence for all the score-impacting questions. The “evidence requests” used until then covered just a third of the score-impacting questions (quality requirements). The case study also informed translation of the quality assessment results in an A, B, C, D, or E score. A weighted score ≥90% resulted in an A, ≥80% resulted in a B, ≥70% resulted in a C, ≥60% resulted in a D, and <60% resulted in an E (Multimedia Appendix 10). Experiences from the telephone calls with manufacturers in which the questions were clarified were used to make the quality assessment framework self-explanatory.

### ISO Procedure

Feedback on the working drafts and editorial remarks in the final vote were used to fine-tune and finalize the TS, including its quality assessment framework. CEN-CENELEC, ISO, and IEC approved the publication of the TS in a near-perfect vote (Multimedia Appendix 11). The TS was published in July 2021 as CEN-ISO/TS 82304-2:2021 Health software – Part 2: Health and wellness apps – Quality and reliability.

### Final Quality Assessment Framework

The final quality assessment framework is included in Multimedia Appendix 8.

## Discussion

This Delphi study aimed to create a useful, globally applicable, trustworthy, and usable health app quality assessment framework.

### Usefulness

To determine its usefulness and overall quality, we compared the CEN-ISO/TS 82304-2 framework with the 20 existing app assessment frameworks evaluated by the World Health Organization (WHO) in 2018 [9]. The TS was found to outqualify all 20 frameworks on all the evaluation criteria used by the WHO, as it (1) addresses all 13 quality domains distinguished by the WHO and adds ethics; (2) includes 4 additional stakeholder perspectives (consumers and patients, insurers, app stores, and app assessors) and thus addresses the continuum of app development and implementation, which none of the frameworks did; and (3) was built on a considerably wider range of existing assessment frameworks and standards.

### Global Applicability

Concerning global applicability, the TS was the result of a standardization effort of 3 prominent international standardization organizations—CEN-CENELEC, ISO, and IEC. In addition, it received a near-perfect vote from these organizations. The project team and Delphi respondents spanned 4 and 6 continents, respectively, although with a predominant representation of Europe, perhaps aligning with the EU’s growing, global relevance in international regulatory affairs and particularly in the digital economy as well as consumer health and safety [27]. Voting members included 6 of the 10 most populous countries worldwide (China, India, the United States, Pakistan, Brazil, and Russian Federation). Of the remaining 4 top-10 countries, Indonesia, Nigeria, and Mexico are observing members of either the IEC or ISO TC. Bangladesh is neither a participating nor an observing member [28]. The quality assessment framework provides a global fit as individual countries, regions, and organizations can set their own profiles for apps, meaning their own thresholds for the uptake of apps in medical guidelines, care contracts, or care pathways with the information provided in CEN-ISO/TS 82304-2’s health app quality report.

### Trustworthiness

Concerning trust, in April 2020, the European Commission referenced CEN-ISO/TS 82304-2 in its EU Toolbox for COVID-19–tracing apps [29]. In June 2021, the Commission launched a Horizon Europe Coordination and Support Action call to promote the adoption of the TS. The 2-year Label2Enable project was selected and started in June 2022 [30]. The Dutch Ministry of Health was the first to request a national health app assessment framework based on the TS. This framework was finalized in May 2021 and presented to Parliament in December 2021 [31]. Starting June 2021, the TS is referenced in Italy as mandatory [32]. The Standing Committee of European Doctors, which represents national medical associations across Europe, proposed in its response to the draft European Health Data Space Regulation to only integrate certified digital applications in Electronic Health Records. “Certified” is specified as complying with ISO standards, referencing solely ISO/TS 82304-2, and being CE-approved [33].

Elements determining the trustworthiness will likely include the evidence base of the TS, specifically the outcomes of the Delphi study with 83 experts, the third-party assessment, requesting evidence for all score-impacting questions, and the upcoming certification scheme. This scheme specifies accreditation requirements for app assessment organizations, what the assessment process of the health app evidence provided entails, when the evidence is deemed sufficient, and when an app requires reassessment. Legislation may be considered as a next step for making the health app quality label widely available and further adding to trust.

### Usability

Concerning usability, CEN-ISO/TS 82304-2’s final quality assessment framework has 81 questions, of which 67 (83%) impact the health app quality score. The case study proved that the quality assessment framework documentation is largely self-explanatory, and the required time investment was acceptable for the app manufacturers involved. The Label2Enable project will work with 6 app assessment organizations from 6 countries and 24 health app manufacturers to test and fine-tune the ISO 17000 series certification scheme it will develop for the TS. In the process, the consistency of the assessments will be evaluated and advanced to promote cross-country recognition of CEN-ISO/TS 82304-2’s quality assessments. Efficiency for both app assessors and app manufacturers will be measured and progressed to enhance the affordability and scalability of CEN-ISO/TS 82304-2 app assessments. A recent article revealed even the national schemes that are front-runners struggle with efficient implementation [11]. Increasing numbers of assessments will likely promote assessment efficiency further, for example, by automating the assessment of specific evidence. Unnecessary duplication of work can be avoided if the many stakeholders across geographic territories all adopt the TS as a standard assessment framework. Crucial context-specific questions can be added on top.

### Adoption Considerations

The Delphi study revealed that to increase uptake, trust is of paramount importance. A strength of CEN-ISO/TS 82304-2’s health app quality assessment framework is its third-party assessment of more than just publicly available evidence. Having a third-party assessment does involve costs, which someone will need to pay. If the app manufacturer is expected to pay, that will likely affect their willingness to participate, especially for health apps that are free of charge. The widespread adoption of the TS, or otherwise increasing the benefits for app manufacturers, would assist in tackling this issue. Alternatively, having the stakeholders that benefit most from the deployment of health apps pay or contribute seems a plausible solution.

The TS can also be used without third-party assessment. App manufacturers may use the TS to determine what should be addressed in the development of a particular app. Health care providers, guideline committees, and insurers may use it as a vocabulary to formulate the requirements for the inclusion of a specific type of app in care pathways, clinical guidelines, or care contracts. We expect that these requirements for adoption and more assessments with the TS will result in further fine-tuning of the evidence required and, in time, of the scoring mechanism. The EU Energy label, one of the inspirers of the health app quality label, has adjusted its scoring mechanism regularly since its launch in 1995. The quality requirement questions are also expected to evolve, as assessment frameworks are known to do. Practical experience, including the certification scheme, will evolve and inform the regular revision process of the TS as mandated by ISO, CEN, and IEC procedures to ensure sustainable fit.

### Outlook

The future will reveal if CEN-ISO/TS 82304-2’s health app quality assessment framework becomes the preferred framework; if it increases the further uptake of apps in care pathways, clinical guidelines, and care contracts; and if the health app quality label gets adopted in app stores, app libraries, and trusted patient and clinician facing health websites. It is promising that different organizations are already taking first steps. The Dutch Ministry of Health and health insurers in the Netherlands are preparing a pilot with 10 to 15 apps using the proposed national health app assessment framework based on the TS [31]. As part of the project “safer health apps,” the Norwegian Directorate of Health has tested 5 apps and promotes 2 of these with the label on their national health portal [34]. Health authorities from Italy and Catalonia are involved in the Label2Enable project [35]. Sweden reportedly already uses the TS [11]. The French Ministry of Health highlights the potential of the TS to help harmonize app quality requirements internationally and reduce the proliferation of different assessment systems in different countries [36]. The Label2Enable project engages with several countries in Europe and beyond. Against the backdrop of a near absence of cross-national policies and the development phase of the ISO 17000 series certification scheme, this uptake is promising [10,11]. The Regulatory Affairs Committee of the European Society of Cardiology has an ongoing initiative to explore the possibility to use the TS for app profiling. Contacts with generic app stores have been established to pursue the publication of the health app quality label. This may prevent the admission of health apps based on manufacturer characteristics instead of quality [37]. If the label becomes as widely used as the EU Energy label that inspired it (4 in 5 purchase decisions), it will also expand the health app user base beyond young, highly educated eHealth-literate users [38,39].

### Conclusions

We developed, together with relevant stakeholders, a useful and usable research-based international standard in health app quality assessment. Its quality, approval rate, and early use provide proof of its potential to become the trusted, commonly used global framework as sought by the European Commission and other stakeholders to improve the quality and reliability, uptake, and public funding of health apps. The framework will help health app manufacturers to enhance and efficiently demonstrate the quality of health apps, consumers, and health care professionals to make informed decisions on health apps, and insurers to make reimbursement decisions about health apps. Legislation may be considered as a next step for making the health app quality label widely available and further adding to trust.

## Appendix

Multimedia Appendix 1 Frameworks Used for the Initial Quality Assessment Framework.

Multimedia Appendix 2 Core experts.

Multimedia Appendix 3 CEN-ISO/TS 82304-2’s health app quality label.

Multimedia Appendix 4 Standards referred to in ISO/TS 82304-2’s quality assessment framework documentation.

Multimedia Appendix 5 COVID-19 symptom apps inclusion criteria.

Multimedia Appendix 6 Characteristics of the participants of the Delphi study and follow-up survey.

Multimedia Appendix 7 Results of the Delphi study.

Multimedia Appendix 8 Final quality assessment framework.

Multimedia Appendix 9 Results of the follow-up survey.

Multimedia Appendix 10 Results of the case study COVID-19 symptom apps.

Multimedia Appendix 11 Results of the final vote.

## Abbreviations

CEN-CENELEC: European Committee for Standardization - European Committee for Electrotechnical Standardization
EU: European Union
IEC: International Electrotechnical Commission
ISO: International Organization for Standardization
SDO: Standard Development Organization
TC: technical committee
TS: technical specification
WHO: World Health Organization

## Data Availability

The individual deidentified participant data of the Delphi study and follow-up survey that underlie the results reported in this paper are available to interested research partners on reasonable request to the corresponding author, 5 years after publication. The prerequisite is a data transfer agreement, approved by the legal department of the requesting researcher and the legal department of Leiden University Medical Center.

## References

[1] (2014). Green paper on mobile Health ("mHealth"). European Commission.

[2] (2013). Socio-economic impact of mHealth: An assessment report for the European Union. PwC.

[3] (2018). Infographic Digital Health and Care in the EU. European Commission.

[4] Kondylakis H, Katehakis DG, Kouroubali A, Logothetidis F, Triantafyllidis A, Kalamaras I, Votis K, Tzovaras D (2020). COVID-19 Mobile Apps: A Systematic Review of the Literature. J Med Internet Res.

[5] Brown RCH, Kelly D, Wilkinson D, Savulescu J (2021). The scientific and ethical feasibility of immunity passports. The Lancet Infectious Diseases.

[6] Webster P (2020). Virtual health care in the era of COVID-19. The Lancet.

[7] (2018). Communication from the Commission to the European Parliament, the Council, the European Economic and Social Committee and the Committee of the Regions on enabling the digital transformation of health and care in the Digital Single Market; empowering citizens and building a healthier society. European Commission.

[8] (2018). mHealth: use of appropriate digital technologies for public health: report by the Director-General. World Health Organization.

[9] Bradway M, Årsand E, Antypas K, Hasvold P, Lee J, Wroblewska N (2018). Report on the mHealth Assessment Frameworks.

[10] D2.1 Knowledge Tool 1. Health Apps Assessment Frameworks. mHealth Hub.

[11] Essén A, Stern AD, Haase CB, Car J, Greaves F, Paparova D, Vandeput S, Wehrens R, Bates DW (2022). Health app policy: international comparison of nine countries' approaches. NPJ Digit Med.

[12] Bates DW, Landman A, Levine DM (2018). Health Apps and Health Policy: What Is Needed?. JAMA.

[13] Bradway M, Carrion C, Vallespin B, Saadatfard O, Puigdomènech E, Espallargues M, Kotzeva A (2017). mHealth Assessment: Conceptualization of a Global Framework. JMIR Mhealth Uhealth.

[14] Mathews SC, McShea MJ, Hanley CL, Ravitz A, Labrique AB, Cohen AB (2019). Digital health: a path to validation. NPJ Digit Med.

[15] Petersen C, Adams SA, DeMuro PR (2015). mHealth: Don't Forget All the Stakeholders in the Business Case. Med 2 0.

[16] Llorens-Vernet P, Miró J (2020). Standards for Mobile Health-Related Apps: Systematic Review and Development of a Guide. JMIR Mhealth Uhealth.

[17] O'Hanlon CE, Fischer SH, Bloom EL (2021). The Business Case for Rigorous Evaluation of Mobile Health apps. Health Affairs Blog August 30.

[18] Budd J, Miller BS, Manning EM, Lampos V, Zhuang M, Edelstein M, Rees G, Emery VC, Stevens MM, Keegan N, Short MJ, Pillay D, Manley E, Cox IJ, Heymann D, Johnson AM, McKendry RA (2020). Digital technologies in the public-health response to COVID-19. Nat Med.

[19] Niederberger M, Spranger J (2020). Delphi Technique in Health Sciences: A Map. Front Public Health.

[20] Jünger S, Payne SA, Brine J, Radbruch L, Brearley SG (2017). Guidance on Conducting and REporting DElphi Studies (CREDES) in palliative care: Recommendations based on a methodological systematic review. Palliat Med.

[21] Hasson F, Keeney S, McKenna H (2008). Research guidelines for the Delphi survey technique. J Adv Nurs 2020.

[22] Lewis M, Woodhull J (2008). Inside The NO: Five Steps to Decisions That Last..

[23] CEN/TC 251 Health Informatics. CEN/TC 251 Health Informatics.

[24] Voting and membership in ISO. ISO.

[25] IEC Electronic Vote & Comment. IEC.

[26] What do patients and carers need in health apps - but are not getting?. PatientView.

[27] Bradford A (2020). The Brussels Effect: How the European Union Rules the World.

[28] (2022). World Population Prospects 2022 Summary of Results. United Nations Department of Economic and Social Affairs.

[29] (2020). Mobile applications to support contact tracing in the EU's fight against COVID-19 - Common EU Toolbox for Member States Version 1. eHealth Network.

[30] (2021). Promoting a trusted mHealth label in Europe: uptake of technical specifications for quality and reliability of health and wellness apps. European Commission.

[31] Blokhuis P (2021). Kamerbrief over adviesrapport over ontwikkeling van toetsingskader voor gezondheidsapps en vervolgproces. Rijksoverheid.

[32] Gara a procedura aperta per la conclusione di un accordo quadro, ai sensi del D.LGS 50/2016 E S.M.I., Avente ad oggetto l'affidamento di servizi applicativi e l'affidamento di servizi di supporto in ambito «SANITA' DIGITALE - Sistemi Informativi Clinico-Assistenziali» per le pubbliche amministrazioni del SSN condizioni della fornitura. consip.

[33] (2022). Position on the European Health Data Space. Standing Committee of European Doctors.

[34] (2022). Tryggere helseapper. Helsedirektoratet.

[35] (2022). Partners. Label2Enable.

[36] (2021). Assessment of apps in the mobile health (mHealth) sector - Overview and quality criteria of medical content for referencing digital services in the digital health space and the professional service package. Haute Autorité de Santé.

[37] Leswing K (2020). Apple is rejecting coronavirus apps that aren’t from health organizations, app makers say. CNBC.

[38] (2019). Europeans' attitudes on EU energy policy. European Commission.

[39] Bol N, Helberger N, Weert JCM (2018). Differences in mobile health app use: A source of new digital inequalities?. The Information Society.

