# Supplementary material for: What Makes a Quality Health App—Developing a Global Research-Based Health App Quality Assessment Framework for CEN-ISO/TS 82304-2: Delphi Study
Source: JMIR Form Res. 2023 Jan 23;7:e43905. doi: 10.2196/43905 (PMC9872976; doi:10.2196/43905)
Supplement: Multimedia Appendix 1 [file formative_v7i1e43905_app1.docx]

**MULTIMEDIA APPENDIX 1**

**Table S1.** Frameworks compared for quality aspects.

| **Frameworks compared for quality aspects** | **Type organization** | **Country** |
| --- | --- | --- |
| Validation pyramid[40] | Healthcare authority | BE |
| MindTech (NHS Choices)[41] | Healthcare authority | GB |
| NHS DAQ[42] | Healthcare authority | GB |
| NICE ESF[43] | Health Technology Assessment (HTA) body | GB |
| ORCHA[44] | App checker | GB |
| PAS277:2015[45] | Standardization | GB |
| UNI TR 11708:2018[46] | Standardization | IT |
| GGD AppStore[47] | Public health | NL |
| Hoofdlijnenakkoord Medisch Specialistische Zorg[48] | Multi stakeholder | NL |
| IGJ Toetsingskader 'Inzet van e-health door zorgaanbieders’[49] | Healthcare authority | NL |
| KNMG Medical App Checker[50] | Healthcare professional | NL |
| National eHealth Living Lab Quick Scan | Academia | NL |
| APA app evaluation framework[51] | Healthcare professional | US |

**Table S2.** Further frameworks used for the initial quality assessment framework.

| **Further frameworks used for the initial quality assessment framework** | **Type organization** | **Country** |
| --- | --- | --- |
| The eHealth Suisse Guide[52] | Healthcare authority | CH |
| Digi-HTA[53] | Academia / Healthcare professional / HTA body | FI |
| NHS Scotland[54] | Healthcare authority | GB |
| Pharos Checklist Toegankelijke informatie[55] | Center of expertise on health disparities | NL |
| Draft code of conduct on privacy for mobile health applications[56] and its official response[57] | App manufacturer | EU |
| EU guidelines on assessment of the reliability of mobile health applications (2^nd^ draft)[58] | Consultancy for (Healthcare) authority | EU |
| Mobile App Rating Scale (MARS)[59] | Academia / Healthcare professional / Manufacturers | AU |
| Xcertia 2019 Board Approved Xcertia Guidelines[60] | Healthcare professional | US |
| HL7 cMHAFF[61] | Standardization | global |
| WHO[62-64] | Healthcare authority | global |

**References**

40. mHealth Belgium. Validation pyramid. 2020; https://mhealthbelgium.be/validation-pyramid.

41. Martin, JL, Simons, L, Craven, MP & Betton, V. Revised Mindtech framework for mental health apps version 1.3. 2015; https://www.researchgate.net/publication/282808875_REVISED_MINDTECH_FRAMEWORK_FOR_MENTAL_HEALTH_APPS_v13_130315.

42. NHS Digital. Digital Assessment Questionnaire V2.1. 2018; https://developer.nhs.uk/wp-content/uploads/2018/09/Digital-Assessment-Questions-V2.1-Beta-PDF.pdf.

43. National Institute for Health and Care Excellence. Evidence Standards Framework for digital health technologies. 2019; https://www.nice.org.uk/Media/Default/About/what-we-do/our-programmes/evidence-standards-framework/digital-evidence-standards-framework.pdf.

44. Organisation for the Review of Care and Health Apps (ORCHA). The ORCHA review. https://www.orcha.co.uk/our-solution/the-orcha-review/#0 Vol. 2019.

45. British Standards Institution (BSI). PAS 277:215 Health and wellness apps. Quality criteria across the life cycle. Code of practice. 2015; https://shop.bsigroup.com/products/health-and-wellness-apps-quality-criteria-across-the-life-cycle-code-of-practice/standard.

46. Ente Italiano di Normazione (UNI). UNI/TR 11708:2018 Informatica Medica - Caratterizzazione delle APP nel contesto della salute, benessere e stili di vita. 2018; http://store.uni.com/catalogo/uni-tr-11708-2018. Italian.

47. Gemeentelijke Gezondheidsdiensten en Geneeskundige Hulpverleningsorganisaties in de Regio (GGD GHOR). GGD AppStore. 2016; https://www.ggdappstore.nl/Appstore/Testmethode (2016).

48. Onderhandelaarsakkoord medisch-specialistische zorg 2019 t/m 2022. 2018; https://demedischspecialist.nl/themas/thema/hoofdlijnenakkoord. Dutch.

49. Inspectie Gezondheidszorg en Jeugd (IGJ). Toetsingskader 'Inzet van e-health door zorgaanbieders’. 2019; https://www.igj.nl/publicaties/toetsingskaders/2019/10/18/toetsingskader-inzet-van-e-health-door-zorgaanbieders. Dutch.

50. Koninklijke Nederlandsche Maatschappij tot bevordering der Geneeskunst. Medical App Checker: Evaluation of Mobile Medical Apps. 2016; KNMG_MedischeApp_170x240_EN.pdf.

51. Torous, JB, Chan, SR, Gipson, SY-MT, Kim, JW, Nguyen, T-Q, Luo, J & Wang, P. A Hierarchical Framework for Evaluation and Informed Decision Making Regarding Smartphone Apps for Clinical Care. Psychiatric Services 2018;69:498-500.

52. Swiss Competence and Coordination Centre of the Confederation and the Cantons. eHealth Suisse Guide for app developers, manufacturers and distributors. 2018; https://www.e-health-suisse.ch/fileadmin/user_upload/Dokumente/2018/E/180731_Leitfaden_fuer_App_Entwickler_def_EN.pdf.

53. Haverinen, J, Keränen, N, Falkenbach, P, Maijala, A, Kolehmainen, T & Reponen, J. Digi-HTA: Health technology assessment framework for digital healthcare services. Finnish Journal of eHealth and eWelfare 2019; 11:326–341.

54. NHS Scotland. Quality Assurance Checklist for Decision Support Resources. https://rightdecision.scot.nhs.uk/quality-assurance-and-regulation

55. Pharos. Checklist Toegankelijke Informatie. https://checklisttoegankelijkeinfo.pharos.nl/checklist. Dutch.

56. Draft Code of Conduct on privacy for mobile health applications. 2017; https://digital-strategy.ec.europa.eu/en/policies/privacy-mobile-health-apps.

57. Article 29 Data Protection Working Party. Subject: your letter of 7^th^ December 2017 and a new draft code of conduct with the request of a positive opinion from the WP29 under the Data Protection Directive. 2018;https://digital-strategy.ec.europa.eu/en/policies/privacy-mobile-health-apps.

58. Ruck, A, Wagner Bondorf, S & Lowe, C. Second draft of guidelines EU guidelines on assessment of the reliability of mobile health applications. 2016; https://ec.europa.eu/health/sites/default/files/ehealth/docs/ev_20160607_co06_04_en.pdf.

59. Stoyanov, SR, Hides, L, Kavanagh, DJ, Zelenko, O, Tjondronegoro, D & Mani, M. Mobile App Rating Scale: A New Tool for Assessing the Quality of Health Mobile Apps. JMIR Mhealth Uhealth 2015;3(1):e27.

60. 2019 Board Approved Xcertia Guidelines. 2019;https://www.himss.org/sites/hde/files/media/file/2020/04/17/xcertia-guidelines-2019-final.pdf.

61. HL7 International. HL7 Consumer Mobile Health Application Functional Framework (cMHAFF), Release 1. https://www.hl7.org/implement/standards/product_brief.cfm?product_id=476 (2018).

62. World Health Organization. Monitoring and evaluating digital health interventions - A practical guide to conducting research and assessment. 2016; https://www.who.int/reproductivehealth/publications/mhealth/digital-health-interventions/en/.

63. World Health Organization. the MAPS Toolkit mHealth Assessment and Planning for Scale. 2015; https://apps.who.int/iris/handle/10665/185238.

64. Agarwal, S, LeFevre, AE, Lee, J, L’Engle, K, Mehl, G, Sinha, C & Labrique, A. Guidelines for reporting of health interventions using mobile phones: mobile health (mHealth) evidence reporting and assessment (mERA) checklist. BMJ 2016;i1174.
