# Supplementary material for: What Makes a Quality Health App—Developing a Global Research-Based Health App Quality Assessment Framework for CEN-ISO/TS 82304-2: Delphi Study
Source: JMIR Form Res. 2023 Jan 23;7:e43905. doi: 10.2196/43905 (PMC9872976; doi:10.2196/43905)
Supplement: Multimedia Appendix 2 [file formative_v7i1e43905_app2.docx]

**Table S3.** Core experts.

| **Core experts** |
| --- |
| *Health app assessment:*  Petra Hoogendoorn (NL)  Matt Leahy (GB) |
| *Standards:*  Charlie McCay (GB)  Norbert Pauli (DE)  Alpo Värri (FI) |
| **Product information** (chapter 5.1 in the TS): Pier Angelo Sottile (IT) |
| **Healthy and safe** (5.2)  Medical devices (5.2.1): Mark Hastenteufel (DE), Koen Cobbaert (BE)  Health risks (5.2.2): Stuart Harrison (GB)  Ethics (5.2.3): Mary Sharp (IE)  Health benefit (5.2.4): Tobias Bonten (NL) Mark Salmon (GB)  Societal benefit (5.2.5): Birgit Morlion (BE) |
| **Easy to use** (5.3)  Accessibility (5.3.1): Anouk Butterlin (NL) Thea Duijnhoven (NL)  Usability (5.3.2): Terhi Holappa (FI) Thea Duijnhoven (NL) |
| **Secure data** (5.4)  Privacy (5.4.1): Marie-José Bonthuis (NL)  Security (5.4.2) Ben Kokx (NL) |
| **Robust build** (5.5)  Technical robustness (5.5.1) and Interoperability (5.5.2) Frank Ploeg (NL) |
