# Supplementary material for: What Makes a Quality Health App—Developing a Global Research-Based Health App Quality Assessment Framework for CEN-ISO/TS 82304-2: Delphi Study
Source: JMIR Form Res. 2023 Jan 23;7:e43905. doi: 10.2196/43905 (PMC9872976; doi:10.2196/43905)
Supplement: Multimedia Appendix 3 [file formative_v7i1e43905_app3.pdf]

Flag or logo

Health app quality label

App icon

App name

Platform icons

Name app manufacturer

Benefit of the app

With this app [intended users] can [intended use] / With this app [x in 10] [intended users] [health effect] [if use]

⚠ Check [here] when app requires approval from a health professional before use

Healthy and safe

B | A

Easy to use

D | C | B | A

Secure data

E | D | C | B | A

Robust build

A

Overall health app quality score

C | B | A

☑ App checked on [date]

CEN-ISO/TS 82304-2:2021
