# Supplementary material for: What Makes a Quality Health App—Developing a Global Research-Based Health App Quality Assessment Framework for CEN-ISO/TS 82304-2: Delphi Study
Source: JMIR Form Res. 2023 Jan 23;7:e43905. doi: 10.2196/43905 (PMC9872976; doi:10.2196/43905)
Supplement: Multimedia Appendix 4 [file formative_v7i1e43905_app4.docx]

MULTIMEDIA APPENDIX 4

1. ISO 9241-210:2019, *Ergonomics of human-system interaction – Part 210 Human-centred design for interactive systems*
2. ISO 14971:2019, *Medical devices – Application of risk management to medical devices*
3. ISO/TR 16982:2002, *Ergonomics of human-system interaction – Usability methods supporting human-centred design*
4. ISO/IEC 27001:2013, *Information technology – Security techniques – Information security management systems – Requirements*
5. ISO 27701:2019, *Security techniques – Extension to ISO/IEC 27001 and ISO/IEC 27002 for privacy information management – Requirements and guidelines*
6. ISO/IEC/TR 29110-1:2016, *Systems and software engineering – Lifecycle profiles for Very Small Entities (VSEs) – Part 1: Overview*
7. ISO/IEC 29184, *Information technology – Online privacy notices and consent*
8. ISO/IEC/IEEE 90003, *Software engineering – Guidelines for the application of ISO 9001:2015 to computer software*
9. IEC 62304:2006, *Medical device software – Software lifecycle processes*
10. IEC 62366-1:2015, *Medical devices – Part 1: Application of usability engineering to medical devices*
11. IEC 82304-1:2016, *Health software – Part 1: General requirements for product safety*
12. HL7 *Consumer Mobile Health Application Functional Framework (cMHAFF),* Release 1
13. AICPA SOC 2 ® – *SOC for Service Organizations: Trust Services Criteria*
14. ANT+. *What is ANT+*
15. Australian Signals Directorate, 2020. Australian Government Information Security Manual (ISM)
16. CREST, 2020. Assurance in Information Security
17. ENISA, 2019. Smartphone Guidelines Tool
18. European Commission, High Level Expert Group on Artificial Intelligence, 2018. Ethics Guidelines for Trustworthy AI
19. Health Information Trust alliance (HITRUST) *HITRUST Common Security Framework*
20. National Institute for Health and Care Excellence (NICE), 2019. *Evidence standards framework for digital health technologies*
21. OWASP *OWASP Mobile Security Testing Guide*
22. OWASP *OWASP Secure Coding Practices – Quick Reference Guide*
23. OWASP 2020. *OWASP Top Ten*
24. Personal Connected Health Alliance *Personal connected health*
25. W3C, 2018. *Web Content Accessibility Guidelines (WCAG) 2.1*
26. ISO/IEC 27017, *Information technology – Security techniques – Code of practice for information security controls based on ISO/IEC 27002 for cloud services*
27. ISO/IEC 27018, *Information technology – Security techniques – Code of practice for protection of personally identifiable information (PII) in public clouds acting as PII processors*
28. ISO 13131, *Health informatics – Telehealth services – Quality planning guidelines*
