# Supplementary material for: What Makes a Quality Health App—Developing a Global Research-Based Health App Quality Assessment Framework for CEN-ISO/TS 82304-2: Delphi Study
Source: JMIR Form Res. 2023 Jan 23;7:e43905. doi: 10.2196/43905 (PMC9872976; doi:10.2196/43905)
Supplement: Multimedia Appendix 5 [file formative_v7i1e43905_app5.docx]

The inclusion criteria for the COVID-19 symptom apps that were invited to use a draft version of the quality assessment framework were:

- apps that inquire about or monitor symptoms for COVID-19.
- apps for smartphone, tablets or websites that are relevant for the Dutch context.
- target group citizens and patients, with or without support by health professionals.
- representative for different functionalities, including self-monitoring symptoms, triage, e-consultation, reporting to health professionals and test results.
