# Supplementary material for: What Makes a Quality Health App—Developing a Global Research-Based Health App Quality Assessment Framework for CEN-ISO/TS 82304-2: Delphi Study
Source: JMIR Form Res. 2023 Jan 23;7:e43905. doi: 10.2196/43905 (PMC9872976; doi:10.2196/43905)
Supplement: Multimedia Appendix 6 [file formative_v7i1e43905_app6.docx]

**MULTIMEDIA APPENDIX 6**

**Table S4.** Characteristics of the participants of the Delphi study and follow-up survey.

|  | **Round 1** | **Round 2** | **Survey** |
| --- | --- | --- | --- |
| **Participants**   - Number of invited participants - Full or partial participation | 197  66 | 211  58 | 76  28 |
| **Description of background**^[[1]](#footnote-1),^^[[2]](#footnote-2)^  1. - App developer / app manufacturer  - SME or industry representative (including digital health promotion, innovation network) | 65:  6  9 | 56:  5  8 | 28:  5 |
| 2. App checker / app assessor (including certification body) | 4 | 2 | 4 |
| 3. App platform | 0 | 3 |  |
| 4. Patient, consumer, or informal caregiver organization (including patient engagement) | 6 | 4 | 5 |
| 5. Medical professional or medical or health professional organization | 9 | 7 | 6 |
| 6. Health insurance (including public funder, funding programme) | 2 | 2 | 1 |
| 7. National or international healthcare authority (including health policy) | 2 | 3 | 7 |
| 8. Academia / subject experts:  - Consultancy  - Standardization expert  - Expert in clinical benefit and/or safety (including ethics and clinical data science)  - Expert in accessibility and/or usability  - Expert in privacy and/or security  - Expert in technical performance and/or interoperability  Undeclared | 4  6  5  2  3  5  2 | 3  4  3  4  3  5  0 | not invited |
| **Primary residence**^[[3]](#footnote-3)^  Africa | 65:  2 | 56:  1 | not asked |
| Asia | 1 | 1 |  |
| Australia | 3 | 2 |  |
| Europe | 53 | 48 |  |
| North America | 2 | 1 |  |
| South America  Undeclared^[[4]](#footnote-4)^ | 0  4 | 1  2 |  |

1. Adjustments were made for 2-round participants. If background was declared once, it was applied to both rounds (n=7). If background differed, nothing was changed. [↑](#footnote-ref-1)
2. In the follow-up survey App developer / app manufacturer and

   SME or industry representative were a combined category. The same applies to

   App assessors and App platforms

   3 Adjustments were made for 2-round participants. If primary residence was declared once, it was applied to both rounds (n=7). If an obvious mistake was made in one of the rounds, it was repaired (n=2). [↑](#footnote-ref-2)
3. 4 In round 1 undeclared consisted likely of Africa (n=1), Australia (n=1), and Europe (n=2), and in round 2 likely solely of Europe (n=2). [↑](#footnote-ref-3)
4. [↑](#footnote-ref-4)
