# Supplementary material for: What Makes a Quality Health App—Developing a Global Research-Based Health App Quality Assessment Framework for CEN-ISO/TS 82304-2: Delphi Study
Source: JMIR Form Res. 2023 Jan 23;7:e43905. doi: 10.2196/43905 (PMC9872976; doi:10.2196/43905)
Supplement: Multimedia Appendix 7 [file formative_v7i1e43905_app7.docx]

**MULTIMEDIA APPENDIX 7**

The quality requirement questions and requests for evidence with a median of less than 6 in rounds 1 and 2 are in light grey, the newly proposed elements are in italic.

**Table S5.** Results of the Delphi study.

| **Round 1** | **Round 2** |
| --- | --- |
| **A. Product information** | |
| **A.1 Product** | |
| A.1.1 What is the health app product name? | A.1.1 What is the name of the health app? |
| A.1.2 What is the health app logo? | A.1.2 What operating system(s) or platform(s) and related version identifier(s) does the health app support? |
| A.1.3 What is the health app platform? | *A.1.3 Is your app only verified for use with specific hardware, browsers, or operating system variants?* |
| A.1.4 What is the current version number of the health app on this platform? | A.1.4 What is the most recent commercial release date and related version identifier of the health app? |
| A.1.5 When was the last release of the health app on this platform? | A.1.5 What is the logo of the health app? |
| A.1.6 Until when are support and updates for the health app guaranteed? | *A.1.6 What is the unique identifier of the health app?* |
| A.1.7 In which countries is the health app on the market? | A.1.7 In which languages is the health app available? |
| A.1.8 In which languages is the health app available on this platform? | A.1.8 What is the web address that provides the information for use of the health app? |
| A.1.9 What is – if applicable – the URL of the (patient) information website of the health app? | To enable app checking, please provide access to the health app |
| EVIDENCE: Access to the health app |  |
| **A.2 App manufacturer** | |
| A.2.1 What is the name of the health app manufacturer? | A.2.1 What is the name of the health app manufacturer? |
| EVIDENCE: Country of registration of the health app manufacturer, registration number (and registration authority) | To enable app checking, please provide country of registration of the health app manufacturer, registration number and registration authority |
| EVIDENCE: Contact person with authority to represent the health app manufacturer | To enable app checking, please provide name, e-mail address and telephone number of the person who is authorized to represent the health app manufacturer |
| **B. Clinical benefit & safety** | |
| **B.1 Medical device** | |
| B.1.1 Is the health app a medical device in one or more of the territories in which the app is on the market? | B.1.1 Has a documented process been followed to establish if the health app is a medical device or in vitro diagnostic medical device in the countries in which the app is on the market? |
| B.1.1.1 Is the health app an in vitro diagnostic medical device? | To enable app checking, please provide evidence of the medical device certification(s) and/or registration(s) or provide the rationale why the health app is not a medical device |
| EVIDENCE: If applicable, copy certification authorized body/bodies |  |
| **B.2 User (health) needs** | |
| B.2.1 Which health issue or health need does the health app target? | *B.2.1 What are the type(s) of intended users?* |
| B.2.2 Can the health issue or health need add to the vulnerability of the intended users of the health app? | B.2.2 Which health issue(s) and/or health need(s) does the health app cover? |
| B.2.3 Can the intended users of the health app include children? | *B.2.3 What type(s) of care does the health app cover?* |
| B.2.4 Was a representation of intended users interviewed to gain an adequate understanding of user (health) needs and current practice prior to making the health app? | B.2.4 What is the minimum age of the intended users? |
| B.2.5 Were qualified health professionals consulted to gain an adequate understanding of user (health) needs and current practice prior to making the health app? | B.2.5 Is the health app a co-creation in which a representative sample of intended users were engaged to establish an adequate understanding of health requirements, contexts and current health interventions? |
| B.2.6 Was scientific evidence used to gain an adequate understanding of user (health) needs and current practice prior to making the health app? | B.2.6 Is the health app a co-creation in which qualified health professionals were engaged to establish an adequate understanding of health requirements, contexts and current health interventions? |
| EVIDENCE: Published evidence user needs | B.2.7 Is the health app informed by peer reviewed scientific literature on intended users’ health requirements, contexts and current health interventions? |
|  | To enable app checking, please provide references to the scientific literature used |
| **B.3 Ethics** | |
| B.3.1 Is the development team of the health app diverse and inclusive? | *B.3.1 Are ethical challenges and risks assessed with intended users and health professionals and adequately addressed in the development of the health app?* |
| B.3.2 Has the development process of the health app been assessed and guided by an external ethics committee? | B.3.2 Are ethical challenges and risks assessed by an impartial and independent ethics advisor or ethics advisory board and adequately addressed in the development of the health app? |
| EVIDENCE: Ethics evaluation methodology and results | *B.3.3 Is an impartial and independent ethics advisor or ethics advisory board involved in reviewing post market surveillance data and follow up measures?* |
|  | *B.3.4 Is evidence available of a positive effect of the health app on health inequalities, access to care for hard-to-reach populations or eliminating discrimination?* |
|  | *To enable app checking, please provide name, e-mail address and telephone number of the impartial and independent ethics advisor or ethics advisory board* |
|  | *To enable app checking, please provide the evidence that the health app has a positive effect on health inequalities, access to care for hard-to-reach populations or eliminating discrimination* |
| **B.4 Health benefit** |  |
| B.4.1 What is the health benefit claimed of using the health app? | B.4.1 What are the claimed health benefit(s) of using the health app? |
| B.4.2 Is evidence available to justify the health app claim? | B.4.1.1 Is the health app capable of delivering the claimed health benefits without additional in-app purchases and/or the purchase of other products or services? |
| B.4.2.1 What level of pre-existing evidence is available? | B.4.1.1.1 Are potential purchasers or users of the health app made aware the health benefit requires in-app purchases and/or the purchase of other products or services? |
| B.4.2.2 Was original research done with the health app to establish the health benefit? | B.4.2 Is evidence available to justify the health app claim? |
| B.4.2.2.1 Did the original research support the claimed health benefit of the health app? | B.4.2.1 Does this evidence include research with this specific health app? |
| B.4.2.2.2 What level of original evidence is available for the health app? | B.4.2.2 What level of evidence is available? |
| B.4.2.2.3 Did a medical ethics board approve the original research with the health app? | *B.4.3 Has a documented process been followed to establish the health risks of using the health app?* |
| B.4.2.3 Is published evidence of the health benefit easily accessible via the health app or website? | *B.4.3.1 Has a documented process been followed to minimize the health risks of using the health app?* |
| B.4.3 Are intended users made aware of the health risks of the health app to enable them to personally weigh pros and cons? | *B.4.3.2 Has a documented process been followed to assess whether the residual risks of using the health app are acceptable?* |
| B.4.4 Are in-app purchases and/or the purchase of other products and services required in order to attain the health benefit? | B.4.3.3 Are potential purchasers or users of the health app given a description of the health risks and side effects of using the health app? |
| B.4.4.1 Is the presence of in-app purchases and/or dependence on other products or services clearly disclosed upfront? | B.4.4 Does the health app make health information available to the user? |
| B.4.5 Does the health app include health information? | B.4.4.1 Is the health information in the health app age-appropriate? |
| B.4.5.1 Is the health information in the health app suitable for vulnerable groups, such as children? | B.4.4.1.1 Are policies in place to appropriately respond to reports of use of the health app by individuals for whom use can be harmful? |
| B.4.5.1.1 Have measures been taken to prevent access to the health app by identified vulnerable groups for whom it is not suitable? | B.4.4.2 Has a documented process been established to ensure the health information in the app is accurate and up to date? |
| B.4.5.2 Has a documented process been established to ensure accuracy of information in the health app? | *B.4.5 Are all sources of funding other than the health app manufacturer disclosed?* |
| B.4.5.3 Does the health app provide promotional content? | B.4.6 Are advertisements clearly distinguishable in the health app? |
| B.4.5.3.1 Is default setting for promotional content opt-in? | *B.4.7 What are the active ingredients of the health app?* |
| B.4.5.3.2 Is promotional content clearly distinguishable in the health app? | B.4.8 Does the health app make clinical judgments? |
| B.4.6 Does the health app include behaviour change techniques? | B.4.8.1 Is the health app intended for use with health professionals? |
| B.4.6.1 Which behaviour change techniques are used in the health app? | B.4.8.2 Have actions been taken to avoid user error and reasonably foreseeable misuse of the health app? |
| B.4.6.2 Is evidence of sustained positive effects available? | B.4.8.3 Does the health app use Artificial Intelligence? |
| B.4.7 Does the health app directly affect clinical care delivery and/or decision-making? | B.4.8.4 Are measures in place to detect and prevent incorrect clinical judgments of the health app? |
| B.4.7.1 Does the health app use adaptive algorithms (AI)? | *B.4.8.5 Is evidence available that the error rate of the app is equal to or better than the average health professional’s error rate?* |
| B.4.7.1.1 Is an ethics by design process used in the development of the AI? | B.4.9 In which condition(s) or with which health issue(s) or symptom(s) are intended users not supposed to use the health app, or to contact a health professional prior to resuming usage of the health app? |
| B.4.7.2 Does the health app apply measures to detect and prevent faulty decision-making and/or faulty clinical care delivery? | B.4.10 Are relevant data on the health benefit and safety of the health app systematically gathered throughout its entire lifetime? |
| B.4.8 Are intended users supported in using the health app by qualified health professionals? | *B.4.10.1 Are benefits and harms for subgroups of intended users analysed?* |
| B.4.9 When are intended users not to use the health app, or to contact a health professional prior to (resuming) usage of the health app? | *B.4.10.2 Is the risk of overdiagnosis monitored?* |
| B.4.10 Are post market surveillance and vigilance procedures used to guide regular improvements to the health benefit risk ratio? | To enable app checking, please provide access to the published research on which the health benefit of the health app is based |
| EVIDENCE: Clinical evidence health benefit | To enable app checking, please provide if applicable evidence of approval of an independent medical ethics board for clinical research |
| EVIDENCE: Approval medical ethics board | To enable app checking, please provide a description of the measures to detect and prevent incorrect clinical judgments by the health app |
| EVIDENCE: Accuracy measures | *To enable app checking, please provide the evidence that the error rate of the app is equal to or better than the average health professional’s error rate* |
| EVIDENCE: Post market surveillance and vigilance procedures |  |
| **B.5 Societal benefit** | |
| B.5.1 Is there a claim that the health app delivers societal benefit? | B.5.1 Is there a claim that the health app delivers societal benefit? |
| B.5.1.1 What is the societal benefit claimed of using the health app? | B.5.1.1 What is the societal benefit claimed of using the health app? |
| B.5.1.2 Is original research available to justify the societal benefit claimed by the health app? | B.5.1.2 Is research with this specific health app available to justify the societal benefit(s) of the health app? |
| B.5.1.2.1 What level of evidence of societal benefit is available for the health app? |  |
| B.5.1.2.2 Is evidence of societal benefit easily accessible via the health app or website? |  |
| B.5.2 Does the health app pose significant financial or organizational risks? |  |
| EVIDENCE: Evidence societal benefit |  |
| **C. Accessibility & usability** | |
| **C.1 Accessibility** | |
| C.1.1 Is the health app WCAG 2.1 AA certified? | C.1.1 Is the health app WCAG 2.1 AA certified or compliant with mobile accessibility guidelines? |
| C.1.1.1 Are information and user interface components of the health app perceivable for all users? | C.1.1.1 Can all intended users perceive all relevant information and user interface components of the health app? |
| C.1.1.2 Are user interface components and navigation of the health app operable for all users? | C.1.1.2 Can all intended users operate all relevant user interface and navigation components of the health app? |
| C.1.1.3 Are user interface and information, in the health app and documents such as instructions for use and privacy statement, understandable for all users? | C.1.1.3 Can all intended users understand all relevant information and user interface components of the health app and related documents, such as terms of service, instructions for use and privacy statement? |
| C.1.1.4 Have actions been taken to avoid use error and reasonably foreseeable misuse of the health app? | *C.1.2 Is the app accessible for persons with low incomes and from resource deprived areas?* |
| C.1.1.5 Is content of the health app robust ie compatible with current and future user agents and assistive technologies? | To enable app checking, please provide evidence of WCAG 2.1 certification or mobile accessibility guidelines compliance |
| C.1.2 Have interface of the health app and elements that require adequate understanding been tested with low health (low tech) literates? |  |
| C.1.3 Is the health app low in consumption? |  |
| C.1.4 Is assured that intermittent network connectivity does not harm the intended user? |  |
| EVIDENCE: WCAG certification |  |
| **C.2 Usability** | |
| C.2.1 Were user requirements and the intended context of use of the health app specified with a representative sample of the intended users? | C.2.1 Is the design based on an explicit understanding of users, tasks and environment? |
| C.2.2 Were scenarios, simulations, models, mock-ups or other types of prototypes for the health app used to gather feedback from the intended users? | C.2.2 Are users involved throughout design and development? |
| C.2.3 Is evidence available that the health app meets user (usability) requirements? | C.2.3 Is the design driven and refined by user-centred evaluation? |
| C.2.4 Are appropriate up-to-date resources available to adequately train users? | *C.2.4 Is the design process iterative?* |
| C.2.4.1 Are patients required to read the instructions for use to adequately use the health app? | *C.2.5 Does the design address the whole user experience?* |
| C.2.5 Are appropriate resources available to adequately help users who experience usability issues? | *C.2.6 Does the health app enable personalization to individual user’s needs and preferences?* |
| C.2.6 Does the post market surveillance plan cover collecting continuous usability feedback to guide regular improvements to usability of both the health app and resources? | C.2.7 Are instructions for use available to adequately instruct users? |
| EVIDENCE: Resources to adequately train users | C.2.7.1 Are users required to see the instructions for use to adequately use the health app? |
| EVIDENCE: Instructions for use | C.2.8 Are appropriate resources available to adequately help users who experience problems using the app? |
|  | C.2.9 Are relevant data on the usability of the health app systematically gathered throughout its entire lifetime, in order to make regular improvements to the usability of the app? |
|  | To enable app checking, please provide access to or point out the instructions for use |
| **D. Privacy & security** | |
| **D.1 Personal data** | |
| D.1.1 Does the health app process personal data? | D.1.1 Does the health app process personal data? |
| D.1.1.1 Does the health app process sensitive personal data? | D.1.1.1 Does the health app process special categories of personal data? |
| D.1.1.2 Does the health app process only necessary data? | D.1.1.2 Is data minimization applied in the health app? |
| D.1.1.3 Is personal data still available to the user if the health app is uninstalled? | D.1.1.3 Is personal data still available to the user if the health app is uninstalled? |
| D.1.1.4 Does the health app have a privacy statement? | D.1.1.4 Is a privacy statement made available to potential purchasers or users of the health app? |
| D.1.1.4.1 Does the privacy statement give a quick simple overview of what the user consent entails? | D.1.1.4.1 Does the privacy statement start with an accessible overview of less than 150 words? |
| D.1.2 Does the health app use cookies? | *D.1.1.4.2 Is consent requested from the holder of parental responsibility for children under the age of 13?* |
| D.1.2.1 Does the health app have a cookie statement? | D.1.2 Does the health app use cookies or trackers besides those strictly necessary for the basic function of the health app? |
| EVIDENCE 1: Privacy statement | D.1.2.1 Does the health app have a cookie statement? |
| EVIDENCE 2: Cookie statement | To enable app checking, please provide access to the privacy statement |
|  | To enable app checking, please provide access to the cookie statement |
| **D.2 Privacy governance** | |
| D.2.1 Is personal data stored elsewhere than just on the device? | D.2.1 Is personal data stored elsewhere than just on the device? |
| D.2.1.1 Is personal data from the health app stored in another jurisdiction and/or processed by third parties? | *D.2.1.1 Are technical and organizational measures in place to govern which persons within the app manufacturer are authorized for which purpose to process the personal data?* |
| D.2.1.1.1 Are written binding contracts in place to enforce whoever wherever involved in the development and operation of the health app and associated services, complies with legislation applicable to the jurisdiction, and applies the same level of security controls and privacy protection as communicated to the user? | D.2.1.2 Is personal data from the health app stored in another jurisdiction and/or processed by third parties? |
| D.2.1.1.2 Is default setting for sharing personal data for commercial reasons opt-in? | D.2.1.2.1 Is default setting for sharing personal data with third parties opt-out? |
| D.2.2 Does the manufacturer have a named individual who is responsible for compliance with and development of documented privacy policies? | D.2.1.2.2 Are data processing agreements in place with all parties in the development and operation of the health app and associated services to ensure the level of security controls and privacy protection are as communicated to the user? |
| D.2.3 Has a data breach-/security-incident response plan been established, that includes reporting data breaches to the intended user and relevant authorities? | D.2.1.3 Does the manufacturer have a Data Protection Officer? |
| EVIDENCE: Name Data Protection Officer | D.2.2 Has a security-incident response plan been established, that includes reporting data breaches to the intended user and relevant authorities? |
|  | To enable app checking, please provide name, e-mail address and telephone number of the Data Protection Officer |
| **D.3 Information security management** | |
| D.3.1 Are the health app manufacturer and the organizations providing associated services certified for ISO 27001? | D.3.1 Are the information security management system of the health app manufacturer and all organizations providing associated services certified for ISO 27001? |
| D.3.2 Is a documented risk management framework used? | *D.3.2 Do risk assessments guide information security measures to ensure a level of security appropriate to the risk of the health app?* |
| D.3.3 Is Application Threat Modelling used as of early in the health app development process to assess security threats? | *D.3.3 Is the security of the health app and associated services tested at major changes and on a regular basis to assess the effectiveness of technical and organizational measures for ensuring confidentiality, integrity and availability?* |
| D.3.4 Does the health app manufacturer PEN-test the health app on a regular basis and at major changes? | D.3.4 Is a documented monitoring process followed for receiving, identifying, assessing, disclosure and timely repair of newly discovered security vulnerabilities? |
| D.3.5 Does a certified third-party PEN-test the health app on a regular basis and at major changes? | To enable app checking, please provide evidence of the ISO 27001 certification |
| D.3.6 Is monitoring for and ensuring timely repair of security vulnerabilities a documented operational process? | To enable app checking, please provide security testing interval, and evidence of third party security testing or certification of the health app |
| D.3.7 Are Coordinated Vulnerability Disclosure (CVD) policies communicated and processes established? |  |
| EVIDENCE 1: ISO 27001 Certification(s) |  |
| EVIDENCE 2: PEN-testing interval and certified third-party |  |
| EVIDENCE 3: CVD website |  |
| **D.4 Health app security** | |
| D.4.1 Is the health app developed using defined secure by design best practices? | D.4.1 Is a documented secure by design process followed? |
| D.4.2 Is the health app developed using defined secure coding best practices? | D.4.2 Is a documented secure coding standard followed? |
| D.4.3 Is the health app developed using the security best practices as defined by the platform? | D.4.3 Is the health app developed using the security best practices as defined by the operating system(s) or platform(s)? |
| D.4.4 Is access to the health app secured by using authentication, authorization, and session management? | D.4.4 Is access to the health app secured by using authentication, authorization, and session management? |
| D.4.4.1 Does the health app have unique credentials per instance? | D.4.5 Does the health app transmit and store all personal and other sensitive data with adequate encryption? |
| D.4.4.2 Does the health app contain any default credentials which are not made unique after installation? | D.4.6 Does the health app gather data from the user and/or use data from other sources? |
| D.4.5 Does the health app transmit and store all personal and other sensitive data with adequate encryption? | D.4.6.1 Does the health app validate all data input via user interfaces or transferred via APIs? |
| D.4.6 Does the health app gather data from the user and/or use data from other sources? | D.4.7 Are all third-party software libraries and other software components used for the health app trusted? |
| D.4.6.1 Does the health app validate all data input via user interfaces or transferred via APIs? | D.4.8 Are all the health app software modules signed? |
| D.4.7 Are all third party software libraries and other software components used for the health app trusted? | D.4.9 Has a documented process been established to prevent unauthorized access and modifications to the health app source code? |
| D.4.8 Are all the health app software modules signed? |  |
| D.4.9 Has a documented process been established to prevent unauthorized access and modifications to the health app source code? |  |
| **E. Technical performance & interoperability** | |
| **E.1 Technical performance** | |
| E.1.1 Is the health app developed with a software development plan that covers the standards, methods and tools to be used? | E.1.1 Is the health app developed with a software development methodology that covers the standards, methods and tools to be used? |
| E.1.2 Are all functional and non-functional requirements for the health app traceable throughout the app project life cycle? | E.1.2 Are all functional and non-functional requirements, tests and test outcomes traceable throughout the app’s life cycle? |
| E.1.3 Is a configuration management plan established for the health app? | E.1.3 Is a configuration management plan established for the health app? |
| E.1.4 Is a validation and verification plan used for the health app, which includes a testing methodology? | E.1.4 Is a validation and verification plan used for the health app? |
| E.1.5 Has a release and deployment mechanism been established? | E.1.5 Has a release and deployment mechanism been established? |
| E.1.6 Is a documented process established for identifying, tracking, and solving issues in the reliability and performance of the health app? | E.1.6 Is a documented process established for identifying, tracking, and solving issues in the reliability and performance of the health app? |
| E.1.7 Is a documented process established for assessing change requests to the health app? | E.1.7 Is a documented process established for assessing change requests to the health app? |
| E.1.8 Has the health app had downtime or impaired functioning in the last six months that jeopardized the intended use and health benefit of the health app? | E.1.8 Has the health app had downtime or impaired functioning over which the manufacturer had control in the last six months that significantly compromised the intended use of the health app? |
| EVIDENCE: Validation and verification plan? | To enable app checking, please provide the validation and verification plan and report |
| **E.2 Interoperability** |  |
| E.2.1 Does the health app have interfaces with websites, apps or other software? | E.2.1 Does the health app have interfaces with websites, apps or other software? |
| E.2.1.1 Is an open standard format used? | *E.2.1.1 Are potential purchasers or users of the health app informed of which interfaces the health app has?* |
| E.2.1.1.1 Is a terminology standard used? | E.2.1.2 Are potential purchasers or users of the health app able to access the specifications and implementation guides for all the interfaces to websites, apps or other software that the health app has? |
| E.2.2 Does the health app facilitate interfaces with external devices? | E.2.1.3 Are potential purchasers or users of the health app able to access the specifications and implementation guides for the terminology or terminologies that are used for coding information? |
| E.2.2.1 Is an open standard format used for interfaces of the health app with external devices? | E.2.2 Does the health app have interfaces with external devices? |
| EVIDENCE: Specification open standard formats | E.2.2.1 Are potential purchasers or users of the health app able to access the specifications and implementation guides for all the interfaces to external devices that the health app has? |
