# Supplementary material for: What Makes a Quality Health App—Developing a Global Research-Based Health App Quality Assessment Framework for CEN-ISO/TS 82304-2: Delphi Study
Source: JMIR Form Res. 2023 Jan 23;7:e43905. doi: 10.2196/43905 (PMC9872976; doi:10.2196/43905)
Supplement: Multimedia Appendix 8 [file formative_v7i1e43905_app8.docx]

**MULTIMEDIA APPENDIX 8**

In this appendix the final quality requirement questions are documented, including their individual purposes, whether they are a minimum requirement (R) to qualify for a health app quality label and whether they have a weight (1, 2 or 3) in the quality scoring. For each question in the quality assessment framework one or more purposes may apply. These purposes include:

1. Label content (Lc): information the health app quality label that communicates the results of the health app quality assessment needs, e.g., icon and name of the health app, supported platforms, health app manufacturer, and health benefit
2. Colour coding (Cc): impacts the health app quality scores communicated via the health app quality label
3. Requirement level (Rl): explores if questions apply, makes the assessment proportional, e.g., if the app does not process personal data privacy questions do not apply
4. Filtering (Fi): enables search and selection of health apps
5. App assessment (Aa): enables app assessment, that is, evaluation of the health app and evidence provided by the app manufacturer

A coloured cell indicates the purpose applies.

Evidence requirements and further clarification text accompany each quality requirement question in ISO/TS 82304-2. The notes and other relevant content have not been included in this appendix. They are needed to operationalise the framework and can be consulted by purchasing ISO/TS 82304-2.^[[1]](#footnote-1)^

**Table S6.** Final quality assessment framework.

|  | **Purposes and weight**  (R = Required) | | | | |
| --- | --- | --- | --- | --- | --- |
|  | **Lc** | **Cc** | **Rl** | **Fi** | **Aa** |
| **PRODUCT INFORMATION**  **Product** | | | | | |
| 5.1.1.1 Which operating systems or platforms does the health app support? |  |  |  |  |  |
| 5.1.1.2 What is the name of the health app? |  |  |  |  |  |
| 5.1.1.3 Provide the health app icon, if available. |  |  |  |  |  |
| 5.1.1.4 In which languages is the health app available? |  |  |  |  |  |
| 5.1.1.5 Provide instructions for access to the health app for assessment. |  |  |  |  |  |
| **App manufacturer** | | | | | |
| 5.1.2.1 What is the name of the health app manufacturer? |  |  |  |  |  |
| 5.1.2.2 Provide e-mail address and telephone number of the person who is authorized to represent the health app manufacturer. |  |  |  |  |  |
| **HEALTHY AND SAFE**  **Health requirements** | | | | | |
| 5.2.1.1 Who are the intended users of the health app? |  |  |  |  |  |
| 5.2.1.2 Are age restrictions of the intended users or subjects of care made clear to potential customers and users? |  | 1 |  |  |  |
| 5.2.1.3 For which health issue(s) and/or health need(s) is the health app intended? |  |  |  |  |  |
| 5.2.1.4 What is the intended use or purpose of the health app? |  |  |  |  |  |
| 5.2.1.5 Are assessments done to establish whether the health app is a medical device or in vitro diagnostic medical device, and if applicable is regulatory approval obtained before the app is made available in each country? |  | 3 |  |  |  |
| 5.2.1.6 Are health professionals involved in the development of the health app? |  | 3 |  |  |  |
| 5.2.1.7 Is appropriate peer reviewed scientific literature used in the development of the health app? |  | 2 |  |  |  |
| **Health risks** | | | | | |
| 5.2.2.1 Are the health risks of the health app analysed? |  | R |  |  |  |
| 5.2.2.2 Are measures used to control the health risks of the health app? |  | 1 |  |  |  |
| 5.2.2.3 Are the residual risks of using the health app found to be acceptable? |  | 1 |  |  |  |
| 5.2.2.4 Describe when the health app requires approval from a health professional before use. |  |  |  |  |  |
| 5.2.2.5 Are potential customers and users of the health app made aware of the health risks, contra-indications, and limitations of use? |  | R |  |  |  |
| 5.2.2.6 Is a process to collect and review safety concerns and incidents for the health app maintained? |  | 3 |  |  |  |
| **Ethics** | | | | | |
| 5.2.3.1 Are ethical challenges of the health app assessed and documented with intended users and health professionals? |  | 1 |  |  |  |
| 5.2.3.2 Is the health app approved by an independent ethics advisor or ethics advisory board? |  | 1 |  |  |  |
| **Health benefit** | | | | | |
| 5.2.4.1 Describe the health benefit of using the app |  |  |  |  |  |
| 5.2.4.2 Are potential customers or users made aware of the health interventions applied to achieve the health benefit? |  | 2 |  |  |  |
| 5.2.4.3 Are potential customers or users made aware of all financial costs to achieve the health benefit? |  | 1 |  |  |  |
| 5.2.4.4 Are potential customers or users made aware of the need for support of a health professional to achieve the health benefit? |  | 2 |  |  |  |
| 5.2.4.5 Is evidence available to support the health benefit of using the app? |  | R* |  |  |  |
| 5.2.4.5.1 Does this evidence include peer reviewed research involving the use of this health app? |  | 1 |  |  |  |
| 5.2.4.5.2 Is the level of the evidence appropriate? |  | 2 |  |  |  |
| 5.2.4.6 Is there a maintenance process for the health information in the app? |  | 1 |  |  |  |
| 5.2.4.6.1 Are all sources for the health information in the health app disclosed to potential customers and users? |  | 2 |  |  |  |
| 5.2.4.7 Are all sources of funding of the health app disclosed to potential customers and users? |  | 1 |  |  |  |
| 5.2.4.8 Is the use of advertising mechanisms disclosed to potential customers and users and are advertisements clearly distinguishable in the health app? |  | 3 |  |  |  |
| **Societal benefit** | | | | | |
| 5.2.5.1 Is evidence available of a societal benefit of using the app? |  | 1 |  |  |  |
| 5.2.5.1.1 Does this evidence include peer reviewed research involving the use of this health app? |  | 1 |  |  |  |
| **EASY TO USE**  **Accessibility** | | | | | |
| 5.3.1.1 Is the health app WCAG 2.1 AA or AAA compliant? |  | 3 |  |  |  |
| 5.3.1.1.1 Are reasonable measures taken to ensure that all intended users can perceive all relevant information and user interface components of the health app and related documents? |  | 1 |  |  |  |
| 5.3.1.1.2 Are reasonable measures taken to ensure that all intended users can operate all relevant user interface and navigation components of the health app and related documents? |  | 3 |  |  |  |
| 5.3.1.1.3 Are reasonable measures taken to ensure that all intended users can understand all relevant information and user interface components of the health app and related documents? |  | 3 |  |  |  |
| 5.3.1.2 Is the health app age-appropriate? |  | 2 |  |  |  |
| **Usability** | | | | | |
| 5.3.2.1 Is the health app design based on an explicit understanding of users, tasks and environment? |  | 2 |  |  |  |
| 5.3.2.2 Are intended users involved throughout design and development of the health app? |  | 2 |  |  |  |
| 5.3.2.3 Is the design of the health app driven and refined by user-centred evaluation? |  | 2 |  |  |  |
| 5.3.2.4 Are measures in place to avoid user error and reasonably foreseeable misuse of the health app? |  | 1 |  |  |  |
| 5.3.2.5 Are potential customers and users provided with adequate product information about the health app? |  | 1 |  |  |  |
| 5.3.2.6 Are instructions for use readily available for users? |  | 3 |  |  |  |
| 5.3.2.7 Are appropriate resources available to adequately help potential customers and users who experience problems with the health app? |  | 1 |  |  |  |
| 5.3.2.8 Are relevant data on the usability of the health app systematically gathered throughout its entire lifetime, in order to make regular improvements? |  | 1 |  |  |  |
| **SECURE DATA**  **Privacy** | | | | | |
| 5.4.1.1 Does the health app process Personally Identifiable Information (PII)? |  |  |  |  |  |
| 5.4.1.1.1 Does the health app process health related PII? |  |  |  |  |  |
| 5.4.1.1.2 Is data minimization applied in the health app? |  | 3 |  |  |  |
| 5.4.1.1.3 Is an appropriate retention policy established to erase or review the data stored? |  | 1 |  |  |  |
| 5.4.1.1.4 Is a privacy statement readily available to potential customers and users of the health app? |  | R** |  |  |  |
| 5.4.1.1.4.1 Does the privacy statement start with an accessible overview in less than 150 words? |  | 3 |  |  |  |
| 5.4.1.1.5 Are contracts in place with all processors and controllers of PII of the health app and associated services to ensure the level of security controls and privacy protection are as communicated to the user? |  | 3 |  |  |  |
| 5.4.1.1.6 Is opt-in the default setting for sharing PII with third parties? |  | 3 |  |  |  |
| 5.4.1.1.7 Does the app manufacturer have a person responsible for legal and regulatory compliance of processing of PII? |  | 1 |  |  |  |
| 5.4.1.1.8 Are security-incident response procedures in place that include reporting PII breaches to the user and relevant authorities? |  | 3 |  |  |  |
| **Security** | | | | | |
| 5.4.2.1 Have the health app manufacturer and all organizations providing associated services implemented and documented the implementation of ISO/IEC 27001? |  | 1 |  |  |  |
| 5.4.2.2 Is an Information Security Risk Assessment documented? |  | 1 |  |  |  |
| 5.4.2.3 Is a secure by design process followed? |  | 3 |  |  |  |
| 5.4.2.4 Are measures in place to ensure that all third-party software libraries and other software components for the health app are reliable and maintained? |  | 1 |  |  |  |
| 5.4.2.5 Is a process to prevent unauthorized access and modifications to the health app source code in place and documented? |  | 2 |  |  |  |
| 5.4.2.6 Are organizational measures in place to ensure PII is processed in a manner that is compatible with the explicit, legitimate purposes specified in the privacy statement? |  | 2 |  |  |  |
| 5.4.2.7 Is user authentication, authorization and session management implemented to secure access to the health app? |  | 1 |  |  |  |
| 5.4.2.8 Does the health app transmit and store all PII with adequate encryption? |  | 1 |  |  |  |
| 5.4.2.9 Are security vulnerabilities reported, identified, assessed, logged, responded to, disclosed, and quickly and effectively resolved? |  | 3 |  |  |  |
| 5.4.2.10 Are the security of the health app and associated services tested on a regular basis and at major changes? |  | 2 |  |  |  |
| 5.4.2.11 Is the information security policy readily available to potential customers and users? |  | 1 |  |  |  |
| **ROBUST BUILD**  **Technical robustness** | | | | | |
| 5.5.1.1 Are all the health app product requirements documented? |  | 1 |  |  |  |
| 5.5.1.2 Is the health app developed with a software development process that covers the standards, methods and tools to be used? |  | 3 |  |  |  |
| 5.5.1.3 Is a secure coding standard followed and documented? |  | 2 |  |  |  |
| 5.5.1.4 Is a configuration management plan established for the health app? |  | 1 |  |  |  |
| 5.5.1.5 Are processes in place to deal with a significant increase or spike in demand? |  | 1 |  |  |  |
| 5.5.1.6 Is a validation and verification plan documented and used for the health app? |  | 3 |  |  |  |
| 5.5.1.7 Is a release and deployment process established? |  | 1 |  |  |  |
| 5.5.1.8 Is a maintenance process established? |  | 3 |  |  |  |
| **Interoperability** | | | | | |
| 5.5.2.1 Are potential customers and users of the health app able to access the specifications and implementation guides for all the APIs? |  | 1 |  |  |  |
| 5.5.2.2 Are potential customers and users of the health app able to access the specifications and implementation guides for the terminology or terminologies used? |  | 1 |  |  |  |
| 5.5.2.3 Does the health app validate all data for the health app transferred via APIs? |  | 1 |  |  |  |
| 5.5.2.4 Can users obtain their PII by a data export to another platform? |  | 1 |  |  |  |

* Depending on intended use

** If the app process Personally Identifiable Information (PII)

**Table S7.** Weight distribution in the ‘Overall health app quality score’.

| **Weight distribution in the ‘Overall health app quality score’** | **Weight** |
| --- | --- |
| Healthy and safe | 5 |
| Easy to use | 1.5 |
| Secure data | 2.5 |
| Robust build | 1 |

1. https://www.iso.org/standard/78182.html (2021). [↑](#footnote-ref-1)
