# Supplementary material for: What Makes a Quality Health App—Developing a Global Research-Based Health App Quality Assessment Framework for CEN-ISO/TS 82304-2: Delphi Study
Source: JMIR Form Res. 2023 Jan 23;7:e43905. doi: 10.2196/43905 (PMC9872976; doi:10.2196/43905)
Supplement: Multimedia Appendix 9 [file formative_v7i1e43905_app9.docx]

**MULTIMEDIA APPENDIX 9**

Participants in the follow-up survey were asked: “Which requirements in the ISO/TS 82304-2 quality assessment are minimum requirements, that is, which questions should in all contexts be answered with yes to qualify for a label?

They were requested to answer in the health app’s intended users’ interest and their contribution to the users’ interest. The user’s best interest includes having transparency in what is a good app and a choice to enable health apps matching individual preferences and budgets.

**Table S8.** Results of the follow-up survey

| **Minimum requirements to qualify for a health app quality label** | **Number of responses** |
| --- | --- |
| 1-2 Are potential customers and users of the health app made aware of the health risks and contra-indications? | 23/27 |
| 1-2 Is evidence available to justify the health app claim? | 23/27 |
| 3-4 Are the health risks of the health app assessed and documented? | 22/27 |
| 3-4 Is a privacy statement readily available to potential customers and users of the health app? | 22/27 |
| 5 Are instructions for use readily available for users? | 20/27 |
| 6-8 Is a process to collect and review safety concerns and incidents for the health app maintained? | 19/27 |
| 6-8 Are security vulnerabilities reported, identified, assessed, logged, responded to, disclosed, and quickly and effectively resolved? | 19/27 |
| 6-8 What is the level of the evidence? | 19/27 |
| 9-10 Are advertisements disclosed to potential customers and users and clearly distinguishable in the health app? | 18/27 |
| 9-10 Are data processing agreements in place with all processors of personal data of the health app and associated services to ensure the level of security controls and privacy protection are as communicated to the user? | 18/27 |

The results of the survey confirmed the 4 minimum requirements to qualify for a health app quality label that resulted in Delphi round 2.
