# Supplementary material for: What Makes a Quality Health App—Developing a Global Research-Based Health App Quality Assessment Framework for CEN-ISO/TS 82304-2: Delphi Study
Source: JMIR Form Res. 2023 Jan 23;7:e43905. doi: 10.2196/43905 (PMC9872976; doi:10.2196/43905)

**MULTIMEDIA APPENDIX 10**

A number of the COVID-19 apps tested were existing apps that were adapted to address COVID-19. Focus was solely on the COVID functionality of the app. To correct for the inability of manufacturers to make use of ISO/TS 82304-2 in the development of their app an adjusted scoring mechanism per question was used. A 50% score could be obtained for individual questions if the requirement was met in part but could be improved. Then the regular scoring mechanism was employed. A weighted score > 90% resulted in an A, > 80% resulted in a B, > 70% resulted in a C, > 60% resulted in a D, and less than 60% resulted in an E. The following scores per app resulted and proved to succeed in distinguishing app quality and reliability in one glance:

**Table S9.** Results of the case study COVID-19 symptom apps.


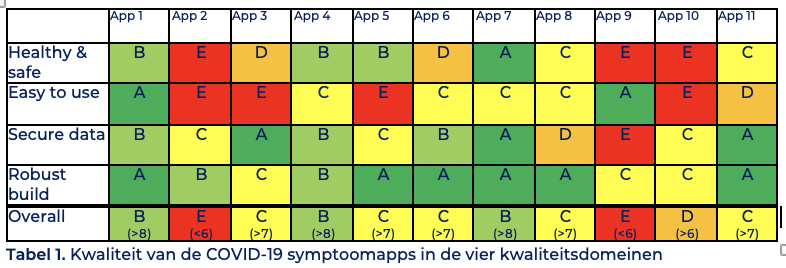

Supplement: Multimedia Appendix 10 [file formative_v7i1e43905_app10.docx]
