# Supplementary material for: What Makes a Quality Health App—Developing a Global Research-Based Health App Quality Assessment Framework for CEN-ISO/TS 82304-2: Delphi Study
Source: JMIR Form Res. 2023 Jan 23;7:e43905. doi: 10.2196/43905 (PMC9872976; doi:10.2196/43905)
Supplement: Multimedia Appendix 11 [file formative_v7i1e43905_app11.docx]

**MULTIMEDIA APPENDIX 11**

**Table S10.** Results of the final vote.

|  | ISO/TC215  Participating member | ISO/TC215  Observing member | CEN/TC251 | IEC/SC 62A  Participating member | IEC/SC 62A Observing member |
| --- | --- | --- | --- | --- | --- |
| Argentina (IRAM) |  |  |  |  |  |
| Armenia (SARM) |  |  |  |  |  |
| Australia (SA)^[[1]](#footnote-1)^ |  |  |  |  |  |
| Austria (ASI) |  |  |  |  |  |
| Bahrain (BSMD) |  |  |  |  |  |
| Belarus (BELST) |  |  |  |  |  |
| Belgium (NBN)^6^ |  |  |  |  |  |
| Brazil (ABNT) |  |  |  |  |  |
| Bulgaria (BDS) |  |  |  |  |  |
| Burundi (BBN) |  |  |  |  |  |
| Canada (SCC) |  |  |  |  |  |
| China (SAC) |  |  |  |  |  |
| Colombia (ICONTEC) |  |  |  |  |  |
| Croatia (HZN) |  |  |  |  |  |
| Cyprus (CYS) |  |  |  |  |  |
| Czech Republic (UNMZ) |  |  |  |  |  |
| Denmark (DS) |  |  |  |  |  |
| Ecuador (INEN) |  |  |  |  |  |
| Egypt (EOS) |  |  |  |  |  |
| Estonia (EVS) |  |  |  |  |  |
| Ethiopia (ESA) |  |  |  |  |  |
| Finland (SFS) |  |  |  |  |  |
| France (AFNOR) |  |  |  |  |  |
| Germany (DIN) |  |  |  |  |  |
| Greece (NQIS/ELOT) |  |  |  |  |  |
| Hong Kong SAR^[[2]](#footnote-2)^ of China (ITCHKSAR) |  |  |  |  |  |
| Hungary (MSZT) |  |  |  |  |  |
| Iceland (IST) |  |  |  |  |  |
| India (BIS) |  |  |  |  |  |
| Indonesia (BSN) |  |  |  |  |  |
| Iran, Islamic Republic of (INSO) |  |  |  |  |  |
| Ireland (NSAI) |  |  |  |  |  |
| Israel (SII) |  |  |  |  |  |
| Italy (UNI) |  |  |  |  |  |
| Japan (JISC) |  |  |  |  |  |
| Kazakhstan (KAZMEMST) |  |  |  |  |  |
| Kenya (KEBS) |  |  |  |  |  |
| Korea, DPR of  (CSK)^[[3]](#footnote-3)^ |  |  |  |  |  |
| Korea, Republic of (KATS) |  |  |  |  |  |
| Latvia (LVS) |  |  |  |  |  |
| Lithuania (LST) |  |  |  |  |  |
| Luxembourg (ILNAS) |  |  |  |  |  |
| Malaysia (DSM) |  |  |  |  |  |
| Malta (MCCAA) |  |  |  |  |  |
| Mexico (DGN) |  |  |  |  |  |
| Mongolia (MASM) |  |  |  |  |  |
| Montenegro (ISME) |  |  |  |  |  |
| Netherlands (NEN) |  |  |  |  |  |
| New Zealand (NZSO) |  |  |  |  |  |
| Nigeria (SON) |  |  |  |  |  |
| North Macedonia, Republic of (ISRSM) |  |  |  |  |  |
| Norway (SN) |  |  |  |  |  |
| Pakistan (PSQCA) |  |  |  |  |  |
| Peru (INACAL) |  |  |  |  |  |
| Philippines (BPS) |  |  |  |  |  |
| Poland (PKN) |  |  |  |  |  |
| Portugal (IPQ) |  |  |  |  |  |
| Romania (ASRO) |  |  |  |  |  |
| Russian Federation (GOST R) |  |  |  |  |  |
| Saudi Arabia (SASO) |  |  |  |  |  |
| Serbia (ISS) |  |  |  |  |  |
| Singapore (SSC) |  |  |  |  |  |
| Slovakia (UNMS SR) |  |  |  |  |  |
| Slovenia (SIST) |  |  |  |  |  |
| South Africa (SABS) |  |  |  |  |  |
| Spain (UNE) |  |  |  |  |  |
| Sri Lanka (SLSI) |  |  |  |  |  |
| Sweden (SIS) |  |  |  |  |  |
| Switzerland (SNV) |  |  |  |  |  |
| Thailand (TISI) |  |  |  |  |  |
| Turkey (TSE) |  |  |  |  |  |
| Ukraine (DSTU) |  |  |  |  |  |
| United Kingdom (BSI) |  |  |  |  |  |
| United States (ANSI) |  |  |  |  |  |
| Uruguay (UNIT) |  |  |  |  |  |

|  | Approval |  | Approval with comments |
| --- | --- | --- | --- |
|  | Abstain |  | Disapproval with justification |
|  | Observing member, no vote | |  |

Within ISO/IEC/CEN/CENELEC consensus is measured as a percentage of approval votes (including approval with comments) compared to total approval and disapproval votes. This percentage needs to be at least 55% (CEN) to 66,7% (ISO, IEC). Disapproval votes require a justification to be valid. Actual approval rates were 100% (16/16, CEN), 95.8% (23/24), adjusted for the editorial issue 100% (24/24, ISO) and 95.7% (22/23, IEC).

1. The negative vote of Belgium was due to an editorial issue that was resolved prior to publication. The technical comments behind the negative vote of Australia will be addressed in the revision of ISO/TS 82304-2. [↑](#footnote-ref-1)
2. SAR = Special Administrative Region [↑](#footnote-ref-2)
3. DPR = Democratic People’s Republic [↑](#footnote-ref-3)
